# Predictive model for assessing the prognosis of rhabdomyolysis patients in the intensive care unit

**DOI:** 10.3389/fmed.2024.1518129

**Published:** 2025-01-10

**Authors:** Yaxin Xiong, Hongyu Shi, Jianpeng Wang, Quankuan Gu, Yu Song, Weilan Kong, Jun Lyu, Mingyan Zhao, Xianglin Meng

**Affiliations:** ^1^Department of Critical Care Medicine, First Affiliated Hospital of Harbin Medical University, Harbin, Heilongjiang, China; ^2^Department of Clinical Research, The First Affiliated Hospital of Jinan University, Guangzhou, China; ^3^Heilongjiang Provincial Key Laboratory of Critical Care Medicine, Harbin, Heilongjiang, China; ^4^Department of Nuclear Medicine, Cancer Institute, Fudan University Shanghai Cancer Center, Shanghai, China

**Keywords:** rhabdomyolysis, intensive care unit, prognosis, nomogram, model

## Abstract

**Background:**

Rhabdomyolysis (RM) frequently gives rise to diverse complications, ultimately leading to an unfavorable prognosis for patients. Consequently, there is a pressing need for early prediction of survival rates among RM patients, yet reliable and effective predictive models are currently scarce.

**Methods:**

All data utilized in this study were sourced from the MIMIC-IV database. A multivariable Cox regression analysis was conducted on the data, and the performance of the new model was evaluated based on the Harrell’s concordance index (C-index) and the area under the receiver operating characteristic curve (AUC). Furthermore, the clinical utility of the predictive model was assessed through decision curve analysis (DCA).

**Results:**

A total of 725 RM patients admitted to the intensive care unit (ICU) were included in the analysis, comprising 507 patients in the training cohort and 218 patients in the testing cohort. For the development of the predictive model, 37 variables were carefully selected. Multivariable Cox regression revealed that age, phosphate max, RR mean, and SOFA score were independent predictors of survival outcomes in RM patients. In the training cohort, the AUCs of the new model for predicting 28-day, 60-day, and 90-day survival rates were 0.818 (95% CI: 0.766–0.871), 0.810 (95% CI: 0.761–0.855), and 0.819 (95% CI: 0.773–0.864), respectively. In the validation cohort, the AUCs of the new model for predicting 28-day, 60-day, and 90-day survival rates were 0.840 (95% CI: 0.772–0.900), 0.842 (95% CI: 0.780–0.899), and 0.842 (95% CI: 0.779–0.897), respectively.

**Conclusion:**

This study identified crucial demographic factors, vital signs, and laboratory parameters associated with RM patient prognosis and utilized them to develop a more accurate and convenient prognostic prediction model for assessing 28-day, 60-day, and 90-day survival rates.

**Implications for clinical practice:**

This study specifically targets patients with RM admitted to ICU and presents a novel clinical prediction model that surpasses the conventional SOFA score. By integrating specific prognostic indicators tailored to RM, the model significantly enhances prediction accuracy, thereby enabling a more targeted and effective approach to managing RM patients.

## 1 Introduction

Rhabdomyolysis (RM), a potentially life-threatening severe muscle injury condition, has complex and diverse causes, including but not limited to crush injuries, trauma, strenuous exercise, metabolic disorders, exposure to drugs and toxins, infections, and genetic factors (1–5). Its core pathological mechanism lies in the abnormal release of critical components such as myoglobin, electrolytes, and various enzymes into the blood circulation after muscle cell damage (6). The release of these harmful substances poses a direct threat to multiple vital organ systems throughout the body (7, 8), significantly worsening the patient’s prognosis and endangering their life safety. Therefore, it is crucial to maintain a high level of vigilance for RM and promptly adopt effective intervention measures to mitigate its potentially severe consequences.

The prognosis of RM patients varies significantly. Mild cases may only present with asymptomatic elevations in serum creatine kinase (CK) levels, while severe cases face more severe health challenges, including dramatic spikes in enzyme levels, severe electrolyte imbalances, acute kidney injury, and even life-threatening complications such as renal failure (9, 10). Currently, the main approaches to the diagnosis and treatment of RM involve early identification and supportive therapy. If RM patients, particularly those with severe conditions, can be identified early and treated promptly, their prognosis is generally better (11, 12).

Early identification of RM patients who may face the risk of adverse clinical outcomes during the early course of the disease can facilitate more precise risk stratification and timely preventive interventions. Although various methods have been employed to assess the severity and prognosis of RM patients, models or tools for accurately predicting patient survival rates remain scarce. Traditional assessment methods primarily rely on clinical manifestations and biochemical indicator examinations (13), but these methods are often limited by subjectivity and low accuracy. This study aims to integrate diverse information, including patients’ detailed medical history, vital signs data, and laboratory tests, to develop a nomogram-based prediction model (14). This model is specifically designed to predict the short-term survival rate of rhabdomyolysis patients, with the goal of enhancing the precision and efficiency of clinical decision-making, thereby optimizing patient treatment processes and long-term prognosis.

## 2 Methods

### 2.1 Data source

This study utilized data from the MIMIC-IV 2.0 database, a publicly accessible resource that contains comprehensive clinical information on intensive care unit (ICU) patients from Beth Israel Deaconess Medical Center (BIDMC) between 2008 and 2019 (15, 16). The authors obtained access to the database after completing the required data research training provided by the associated institution. The data were sourced from the official Physionet website^1^.

### 2.2 Patient population

**Figure 1:**
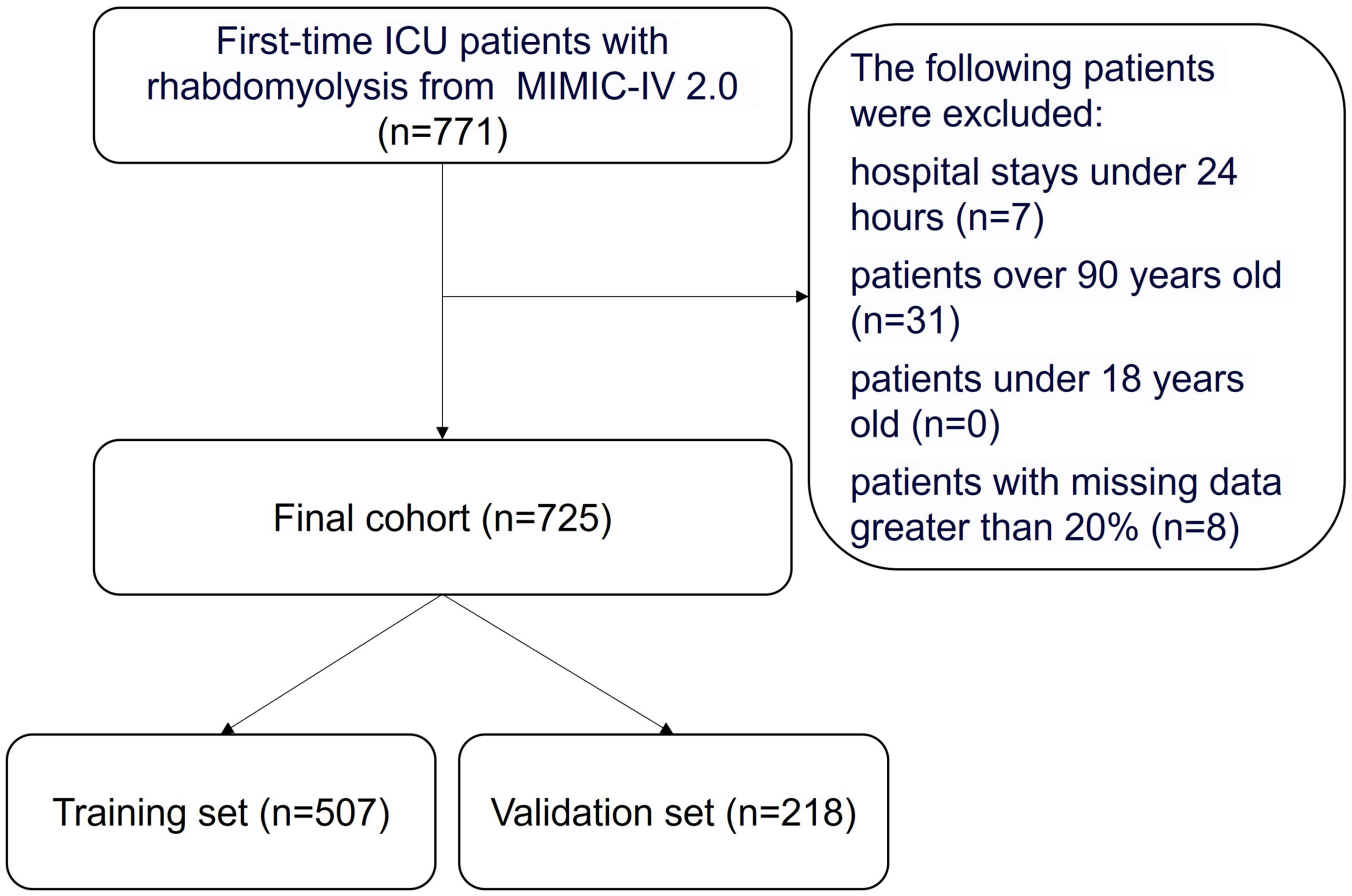
Flowchart of the study. ICU, indicates intensive care unit; MIMIC-IV, Multiparameter Intelligent Monitoring in Intensive Care Database IV.

The ICD-9 standard code 72888 and ICD-10 standard code M6282 were utilized to identify 771 RM patients who were first admitted to the ICU. Patients with hospital stays shorter than 24 h, those aged over 90 or under 18, and patients with more than 20% missing data were excluded. Ultimately, 725 patients who met the inclusion criteria were selected. The process of data selection based on these criteria is illustrated in Figure 1. We randomly assigned 70% of the patients to the training cohort and used the remaining 30% as the validation cohort.

### 2.3 Data extraction

We have not only extracted the basic information of patients but also identified potential variables pertinent to the prognosis assessment of patients with rhabdomyolysis, based on clinical experience and relevant literature (7, 9, 17, 18). These variables encompass physiological indicators, blood tests, comorbidities, and admission assessments. The data were extracted via Structured Query Language (SQL), specifically including patients’ demographic information such as gender, age, and height, along with temporal information including admission time, discharge time, time of death, ICU admission time, ICU discharge time. Comorbidities assessed were hyperlipidemia, hypertension, chronic pulmonary disease, liver disease, diabetes, renal disease, and hypothyroidism. Vital signs and laboratory values on the first day of admission included mean values for HR (heart rate), SBP (systolic blood pressure), DBP (diastolic blood pressure), RR (respiratory rate) and SPO2 (oxygen saturation), as well as mean blood glucose. The minimum values recorded for Hb (hemoglobin), PLT (platelet count), RDW (red cell distribution width), Ca (calcium), and HCO3 (bicarbonate) were also extracted. Additionally, the maximum values for WBC (white blood cell count), AG (anion gap), BUN (blood urea nitrogen), Cr (creatinine), ALT (alanine aminotransferase), ALP (alkaline phosphatase), AST (aspartate aminotransferase), TBIL (total bilirubin), PT (prothrombin time), APTT (activated partial thromboplastin time), CK (creatine kinase), Ca (calcium), Phosphate, and K (potassium) were included. Urine output and the first SOFA (Sequential Organ Failure Assessment) score on admission were also part of the extracted data.

### 2.4 Outcome

We have identified the all-cause mortality rates at 28 days, 60 days, and 90 days post-admission as the outcomes of our study.

### 2.5 Statistical analysis

The detailed information on missing data is summarized in Supplementary Table S1. Notably, no missing values were observed for categorical variables, while some continuous variables exhibited random missing data. However, the proportion of missing data for all variables was less than 15%. We employed the MICE package in R software, utilizing Predictive Mean Matching (PMM) within the multiple imputation framework to address these missing values (19). Additionally, we visually presented the distribution of data before and after imputation (Supplementary Figure S1) and conducted descriptive statistical analyses on the data both before and after imputation (Supplementary Table S2).

In this study, continuous variables that satisfied the normal distribution were represented by the mean ± standard deviation (SD), whereas those that did not conform to a normal distribution were expressed using the median and quartiles [M (Q1, Q3)]. Categorical variables were presented as frequencies and percentages. Comparisons between continuous variables were performed using the Wilcoxon rank-sum test, while comparisons among categorical variables were conducted using Fisher’s exact test.

In the training cohort, an initial univariate analysis was conducted to identify differences between groups and select potential prognostic indicators. Subsequently, a multivariate Cox regression model was employed to further analyze the variables that exhibited significant differences in the univariate analysis (20). After selecting the variables determined to be significant, the variance inflation factor (VIF) was calculated as an indicator of collinearity analysis. The results indicated that all variables had VIF values less than 2, suggesting that there were no significant collinearity issues among the variables in our dataset. These significant variables were then utilized to develop nomograms for predicting the 28-day, 60-day, and 90-day survival rates of patients with RM (21). The predictive accuracy of these nomograms was evaluated using the Harrell concordance index (C-index) and the area under the receiver operating characteristic curve (AUC). Calibration curves were employed to assess the agreement between predicted probabilities and actual outcomes, while decision curve analysis (DCA) was conducted to test the clinical utility of the prediction models (22). The performance of the prediction models was benchmarked against the Sequential Organ Failure Assessment (SOFA) score. The net reclassification improvement (NRI) was utilized to compare the accuracy of the two models, and the integrated discrimination improvement (IDI) was calculated to determine the effectiveness of the improvements (23).

All statistical analyses were conducted using R software, version 4.3.3 (R Foundation for Statistical Computing, Vienna, Austria). All tests were two-sided, and *p*-values <0.05 were considered statistically significant.

## 3 Results

### 3.1 Baseline characteristics of patients

This study encompassed 725 eligible patients with rhabdomyolysis, with 507 patients in the training cohort and 218 patients in the validation cohort. Among these patients, the mortality rates in the training and validation cohorts were 20.51 and 21.56%, respectively. The training cohort comprised 357 males (70.41%) and 150 females (29.59%), with a median age of 55 years (IQR = 41–68 years). The validation cohort consisted of 147 males (67.43%) and 71 females (32.57%), with a median age of 58 years (IQR = 38–72 years) (Table 1).

**Table 1:** Baseline characteristics and outcomes of patients with rhabdomyolysis.

Characteristic	Train, *N* = 507	Validation, *N* = 218	*p*-value
Gender			0.430
Male	357 (70.41%)	147 (67.43%)	
Female	150 (29.59%)	71 (32.57%)	
Age	55.00 (41.00, 68.00)	58.00 (38.00, 72.00)	0.288
Weight	83.40 (70.00, 99.10)	80.00 (67.13, 96.98)	0.061
Hb min	11.00 (9.30, 12.35)	11.10 (9.80, 12.40)	0.219
PLT min	162.00 (110.00, 212.00)	169.50 (109.50, 229.75)	0.306
WBC max	14.10 (10.25, 18.60)	13.40 (10.20, 17.10)	0.100
RDW	13.90 (13.20, 15.00)	14.00 (13.20, 14.78)	0.856
AG max	19.00 (16.00, 23.00)	19.00 (15.00, 24.00)	0.787
HCO3 min	19.00 (16.00, 22.00)	19.50 (16.00, 22.00)	0.605
BUN max	28.00 (17.00, 46.00)	27.50 (17.00, 52.00)	0.767
Cr max	1.70 (1.00, 3.10)	1.65 (1.03, 3.20)	0.928
ALT max	77.00 (39.00, 237.00)	64.00 (33.00, 202.00)	0.307
ALP max	76.00 (57.00, 104.00)	76.00 (58.25, 105.50)	0.509
AST max	185.00 (81.00, 570.50)	148.50 (71.25, 508.00)	0.251
TBIL max	0.70 (0.40, 1.20)	0.70 (0.40, 1.20)	0.791
PT max	13.80 (12.50, 16.75)	13.60 (12.20, 16.70)	0.245
APTT max	31.90 (27.30, 42.75)	30.15 (27.10, 38.60)	0.034
CK max	5,363.00 (1,855.00, 14,301.50)	4,927.00 (1,972.50, 14,721.00)	0.736
Ca min	7.60 (6.90, 8.10)	7.60 (7.13, 8.20)	0.536
Ca max	8.30 (7.90, 8.90)	8.40 (7.90, 8.98)	0.418
Phosphate max	4.00 (3.10, 5.40)	4.00 (3.00, 5.30)	0.579
K max	4.60 (4.10, 5.50)	4.50 (4.00, 5.28)	0.248
HR mean	90.00 (80.00, 102.00)	91.00 (81.00, 104.00)	0.304
SBP mean	116.00 (106.50, 130.00)	119.00 (108.00, 132.75)	0.170
DBP mean	65.00 (59.00, 74.00)	67.00 (60.00, 75.00)	0.306
RR mean	20.00 (17.00, 23.00)	20.00 (17.00, 22.00)	0.745
SPO2 mean	97.00 (96.00, 99.00)	97.00 (96.00, 98.00)	0.231
Blood glucose mean	125.50 (103.00, 155.50)	124.50 (99.05, 155.73)	0.406
Urine output	1,610.00 (718.50, 2,790.50)	1,670.00 (892.50, 2,747.50)	0.412
Hyperlipidemia	118 (23.27%)	49 (22.48%)	0.848
Hypertension	194 (38.26%)	87 (39.91%)	0.679
Chronic pulmonary disease	107 (21.10%)	42 (19.27%)	0.617
Liver disease	126 (24.85%)	42 (19.27%)	0.104
Diabetes	120 (23.67%)	52 (23.85%)	>0.999
Renal disease	70 (13.81%)	34 (15.60%)	0.564
Hypothyroidism	46 (9.07%)	22 (10.09%)	0.678
SOFA	6.00 (4.00, 10.00)	6.00 (3.00, 9.00)	0.118
LOS ICU	3.00 (2.00, 7.00)	3.00 (2.00, 6.00)	0.320
LOS hospital	9.00 (6.00, 17.00)	8.00 (5.00, 16.00)	0.143
Mortality rate	104 (20.51%)	47 (21.56%)	0.765

Hb, hemoglobin; PLT, platelet count; WBC, white blood cell count; RDW, red cell distribution width; AG, anion gap; HCO3, carbonic acid hydrogen radical; BUN, blood urea nitrogen; Cr, creatinine; ALT, alanine aminotransferase; ALP, alkaline phosphatase; AST, aspartate aminotransferase; TBIL, total bilirubin; PT, prothrombin time; APTT, activated partial thromboplastin time; CK, creatine kinase; Ca, calcium; K, potassium; HR, heart rate; SBP, systolic blood pressure; DBP, diastolic blood pressure; RR, respiratory rate; SPO2, oxygen saturation.

In the univariate analysis of the training cohort, significant disparities were observed between the survival and non-survival groups regarding age, Hb min, PLT min, WBC max, RDW min, AG max, HCO3 min, BUN max, Cr max, ALT max, ALP max, AST max, TBIL max, PT max, APTT max, phosphate max, K max, SBP mean, DBP mean, RR mean, SPO2 mean, blood glucose mean, urine output, SOFA score, and liver disease (Supplementary Table S3). These notably different variables were recognized as potential prognostic indicators influencing patients’ short-term mortality and were subsequently included in the Cox regression analysis.

### 3.2 Predictors associated with short-term mortality in patients with RM

When developing a predictive model using a multivariate COX regression approach, six predictors were significantly associated with patient mortality: age (HR = 1.045, *p* < 0.001), Phosphate max (HR = 1.166, *p* = 0.009), RR mean (HR = 1.054, *p* = 0.047), and SOFA score (HR = 1.145, *p* < 0.001) (Table 2).

**Table 2:** Multivariate Cox regression analysis.

Characteristic	Hazard Ratio	95% CI	*p*-value
Liver disease			
No	Reference	—	—
Yes	1.082	(0.655, 1.788)	0.757
Age	1.045	(1.028, 1.063)	<0.001
Hb min	1.106	(0.995, 1.229)	0.061
PLT min	0.998	(0.995,1.001)	0.112
WBC max	1.022	(0.994, 1.050)	0.130
RDW	1.079	(0.956, 1.217)	0.218
AG max	1.012	(0.965,1.061)	0.627
HCO3 min	1.002	(0.948, 1.059)	0.952
BUN max	1.005	(0.995, 1.015)	0.309
Cr max	0.851	(0.722, 1.002)	0.053
ALT max	1.000	(1.000,1.000)	0.580
ALP max	1.002	(1.000,1.003)	0.060
AST max	1.000	(1.000,1.000)	0.414
TBIL max	1.004	(0.900, 1.120)	0.939
PT max	1.005	(0.992,1.018)	0.460
APTT max	1.005	(0.999,1.010)	0.094
Phosphate max	1.166	(1.039, 1.309)	0.009
K max	0.994	(0.810, 1.219)	0.953
SBP mean	1.001	(0.984, 1.019)	0.892
DBP mean	1.017	(0.992, 1.043)	0.177
RR mean	1.054	(1.001, 1.110)	0.047
SPO2 mean	0.957	(0.901, 1.017)	0.154
Blood glucose mean	1.002	(0.998,1.005)	0.431
Urine output	1.000	(1.000,1.000)	0.636
SOFA	1.145	(1.080,1.215)	<0.001

### 3.3 Predictive model for survival rates in RM patients

**Figure 2:**
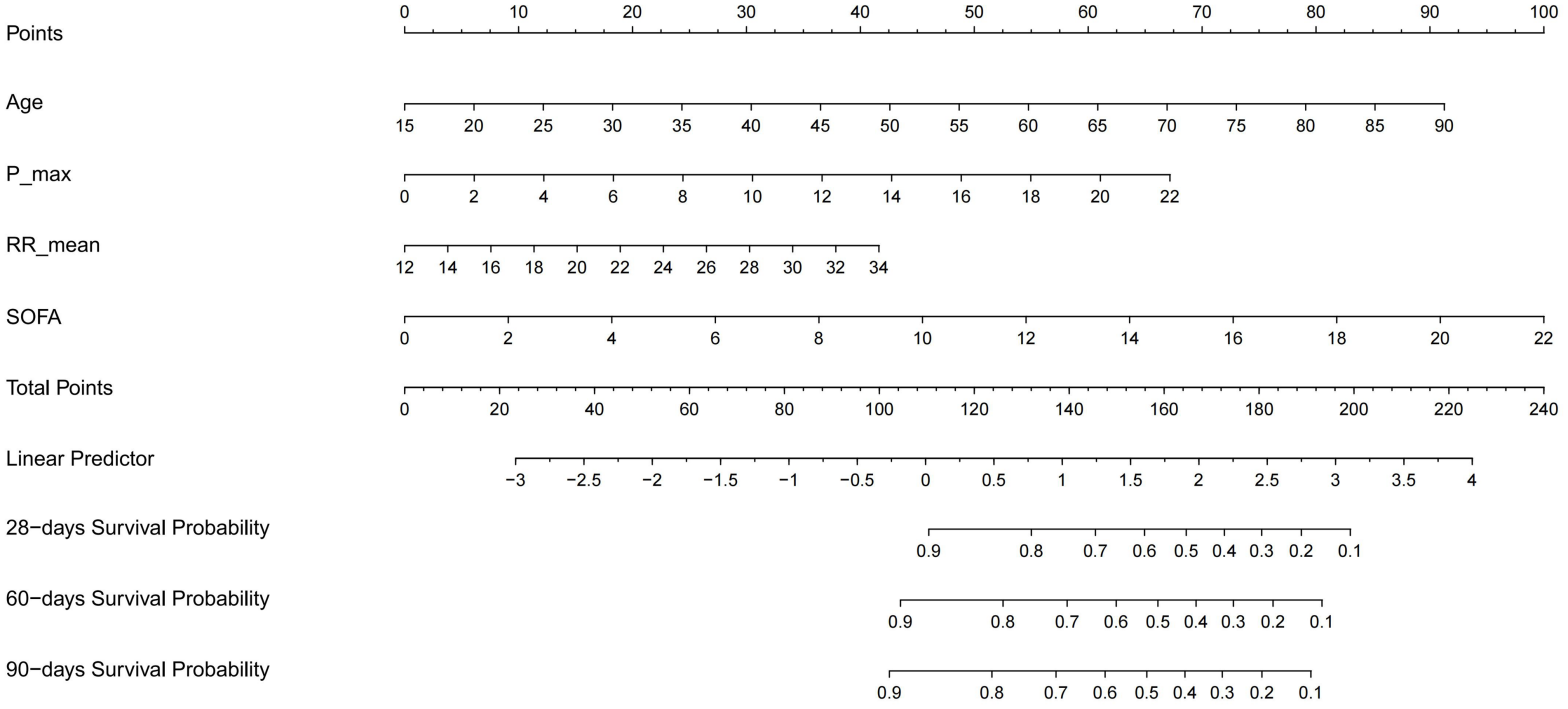
Nomogram predicting 28-, 60-, and 90-day survival rate. Left column shows the points bar (top) and four parameters, each to be scored with a vertical line to the points bar, according to the different parameter values. The sum of the points is calculated (total points range, 0–240), and a vertical line is drawn from the total points bar to the 28−/60−/90-days survival probability below, to obtain survival probability of the patient. RR, respiratory rate; SOFA, Sequential organ failure assessment.

A visual nomogram was developed to predict the survival rates of RM patients in the training cohort (Figure 2). This nomogram incorporates all significant independent factors contributing to survival prediction within the training cohort. Among them, SOFA score as the most critical factor, with additional factors including age, phosphate max and RR mean.

### 3.4 The performance and clinical practicality of the novel model

**Figure 3:**
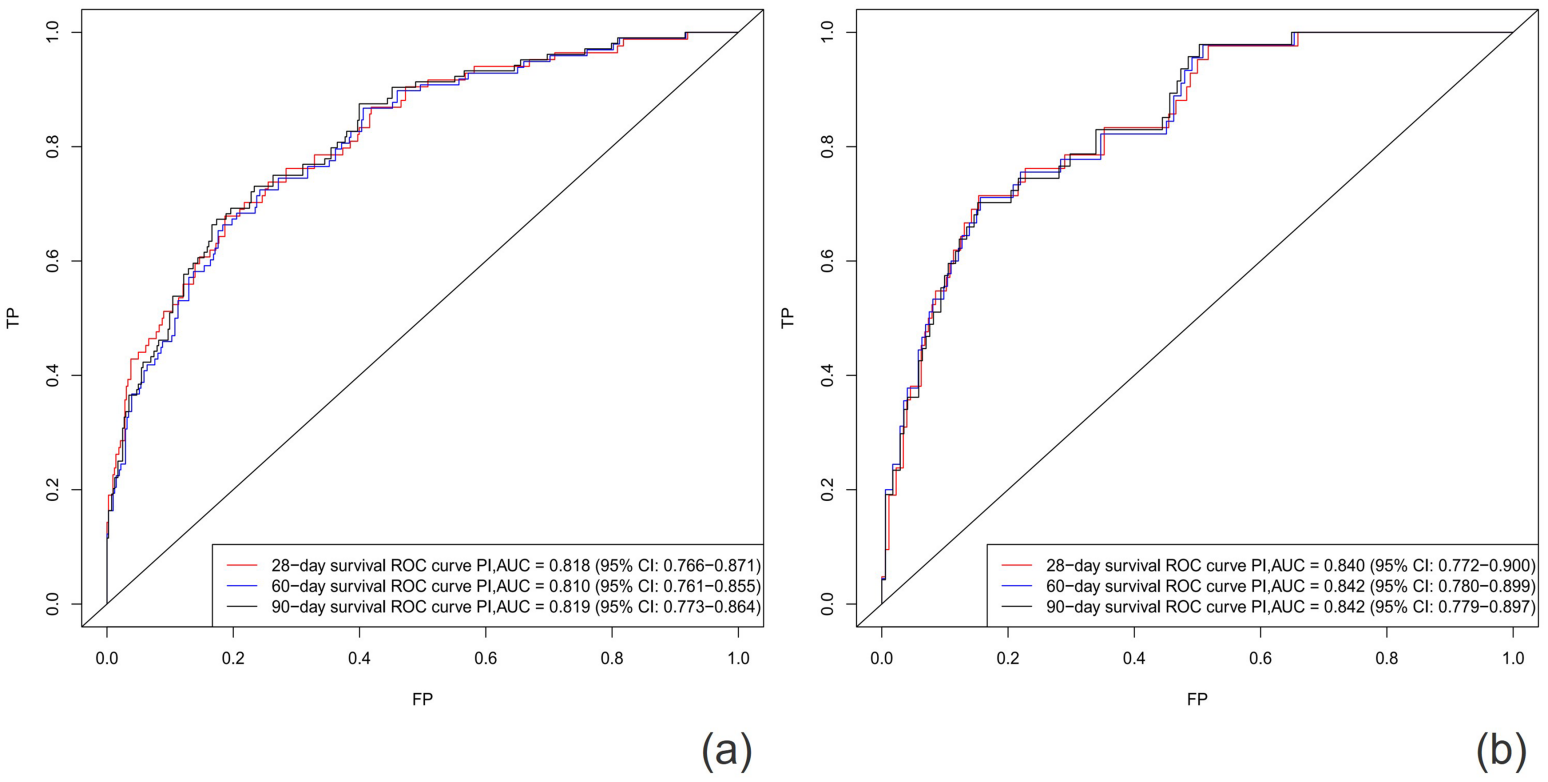
Area under the ROC curves (AUC) of training cohort **(A)** and validation cohort **(B)**.

**Figure 4:**
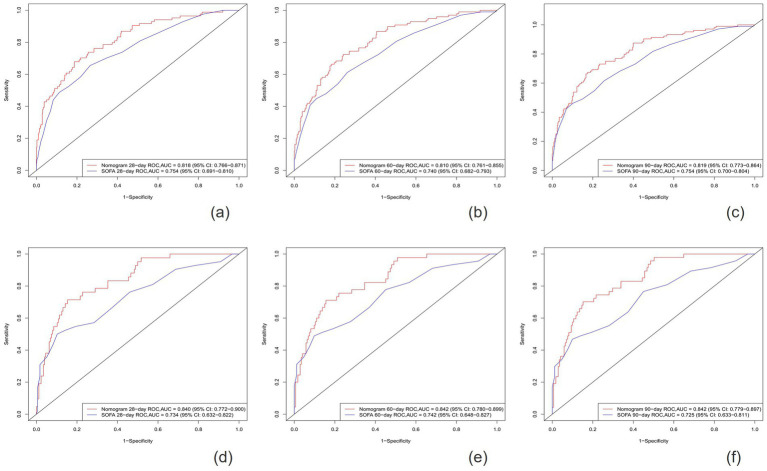
The ROC curves for the Nomogram and SOFA scores are presented for both the training cohort **(A–C)** and the validation cohort **(D–F)**.

After establishing the nomogram, we utilized a series of metrics to evaluate the performance of the novel predictive model derived from it. Our findings revealed that the new model consistently outperformed the SOFA score in terms of the C-index, both in the training cohort (0.795 vs. 0.734) and the validation cohort (0.805 vs. 0.700). Specifically, the AUC values for predicting 28-day, 60-day, and 90-day survival rates in the training cohort were 0.818 (95% CI: 0.766–0.871), 0.810 (95% CI:0.761–0.855), and 0.819 (95% CI: 0.773–0.864), respectively, all exceeding those of the SOFA score [AUC values of 0.754 (95% Cl: 0.691–0.810), 0.740 (95% CI: 0.682–0.793), and 0.754 (95% CI: 0.700–0.804)]. Similarly, in the validation cohort, the new model demonstrated superior predictive performance, with AUC values of 0.840 (95% CI: 0.772–0.900), 0.842 (95% CI: 0.780–0.899), an 0.842 (95% CI: 0.779–0.897) for 28-day, 60-day, and 90-day survival rates, respectively, compared to the SOFA score’s AUC values of 0.734 (95% CI: 0.632–0.822), 0.742 (95% CI: 0.648–0.827), and 0.725 (95% CI, 0.633–0.811) (Figures 3, 4).

**Figure 5:**
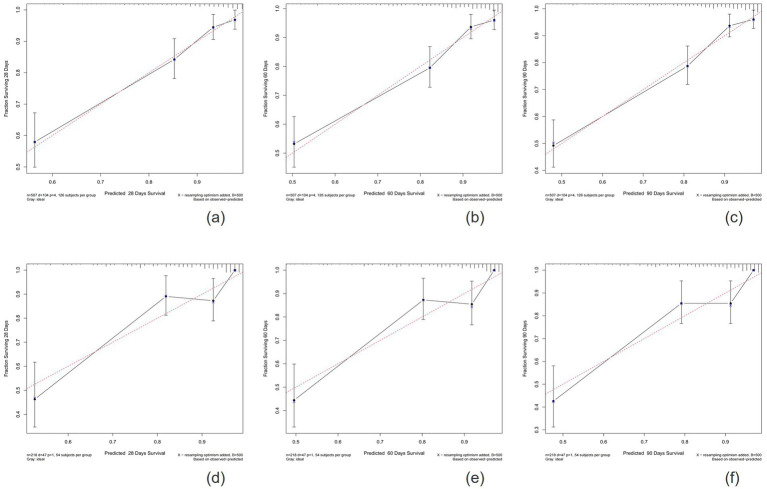
Calibration plots showing the relationship between the predicted probabilities based on the nomogram and actual values of the training cohort **(A–C)** and validation cohort **(D–F)**.

**Figure 6:**
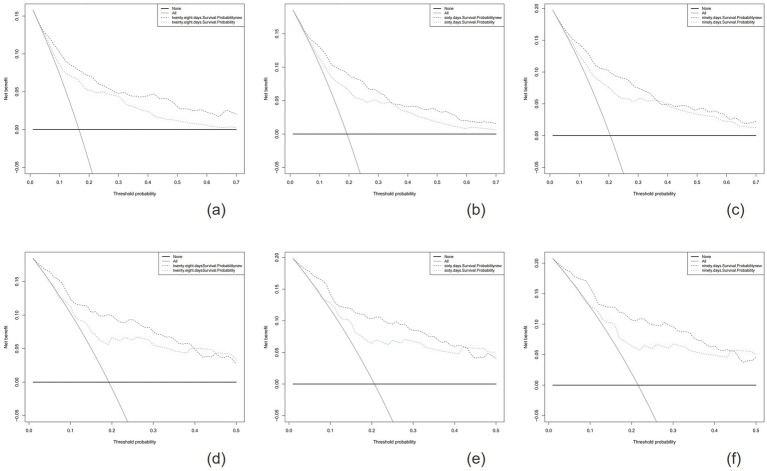
The horizontal axis represents the threshold probability, and the vertical axis represents the net benefit rate. The horizontal line indicates that all samples are negative and no treatment is given. The oblique line indicates that all samples are positive and all are treated. The net benefit is depicted as a backslash with a negative slope. The figures show the results for the training cohort **(A-C)** and the validation cohort **(D-F)**.

The calibration plots indicated that the standard curves for 28-day, 60-day, and 90-day survival rates closely approximated the ideal 45-degree diagonal line, with evenly distributed calibration points, suggesting excellent calibration ability of the new model (Figure 5). The DCA curves, where the horizontal axis represents the threshold probability and the vertical axis represents the net benefit, demonstrated higher net benefits for the new model compared to the SOFA score at 28-day, 60-day, and 90-day intervals in both the training and validation cohorts (Figure 6). When the threshold probability exceeds 0.01, respectively, the use of this nomogram yields higher net benefits for predicting the 28-day, 60-day, and 90-day mortality risks associated with rhabdomyolysis.

### 3.5 Predictive accuracy of the novel model

In the training cohort, the NRI values for 28-day, 60-day, and 90-day survival rates were 0.459 (95% CI: 0.231–0.700), 0.494 (95% CI: 0.259–0.737), and 0.474 (95% CI: 0.246–0.726), respectively. In the validation cohort, the corresponding NRI values were 0.544 (95% CI: 0.041–0.993), 0.520 (95% CI: 0.009–0.944), and 0.565 (95% CI: 0.119–0.999). These results demonstrate that the predictive performance of the novel model surpasses that of the SOFA score. Additionally, in the training cohort, the IDI values for 28-day, 60-day, and 90-day survival rates were 0.080, 0.087, and 0.090, respectively (all *p* < 0.001), while in the validation cohort, the IDI values were 0.089, 0.095, and 0.100, respectively (all *p* < 0.001). The fact that all NRI and IDI values are greater than zero indicates that the novel model exhibits superior discriminative ability compared to the SOFA score.

## 4 Discussion

The RM is a syndrome resulting from skeletal muscle breakdown, characterized by the release of myoglobin, other intracellular proteins, and electrolytes into the bloodstream (17, 24). Studies indicate that the mortality rate among RM patients is approximately 10%, with a significant increase in mortality when acute renal failure occurs (25, 26). Currently, the clinical assessment of disease severity and prognosis in RM patients primarily relies on a comprehensive approach combining symptomatic observation and laboratory tests (9). Furthermore, several studies have been conducted to develop prognostic prediction models for RM patients, aiming to provide more precise assessment tools. Liu et al. (27) focused on early prediction of the need for renal replacement therapy (RRT) in RM patients, providing crucial insights for clinical decision-making. Despite the establishment of two reliable prognostic prediction models, their applicability is relatively limited, primarily focusing on predicting in-hospital mortality risk (9, 28). This implies that, despite the existence of various prognostic prediction models, there remains a need to address the prediction of different prognostic outcomes. To enhance patients’ 90-day survival rates, we have developed a nomogram that clinicians can utilize to analyze patient prognosis and refine treatment strategies.

Using multivariate Cox regression analysis, we identified four variables independently associated with patient mortality: age, phosphate levels, respiratory rate, and SOFA (Sequential Organ Failure Assessment) score. These variables were ultimately utilized to develop a clinical prognostic model. Age, as a demographic factor, has shown significant importance in most medical contexts, consistent with our findings. With advancing age, patients experience gradual physiological decline and decreased resistance, posing threats to their survival (29). In RM patients, extensive muscle cell damage leads to the massive release of intracellular phosphate into the bloodstream (12). Hyperphosphatemia not only increases the risk of acute kidney injury among hospitalized patients but also serves as an independent risk factor for mortality in critically ill patients (30–33). Our results indicate a linear association between phosphate levels and mortality, aligning with previous research (34, 35). Respiratory rate serves as a sentinel, one of the most vital signs, as its normal range is often breached before other vital signs in nearly all cases of clinical deterioration (36). Despite ample evidence suggesting that abnormal respiratory rates are early predictors of patient deterioration, they have not received the attention they deserve (37–39, 41, 42). Our study also demonstrates a correlation between respiratory rate and early mortality in RM patients, further confirming its significance. The SOFA scoring system is a tool for predicting patient prognosis by assessing the functional status of six major organ systems (respiratory, coagulation, liver, cardiovascular, central nervous system, and renal functions) (40). It provides a comprehensive evaluation of various physiological indicators in critically ill patients, with higher scores indicating more severe organ failure and poorer prognosis.

The predictive model developed in this study demonstrates significant clinical application value. Firstly, the variables incorporated in the model are all routine clinical test indicators, which are readily accessible and monitorable. Secondly, while the SOFA score is frequently utilized to predict survival rates among critically ill patients, it fails to comprehensively capture the specific pathophysiological alterations associated with RM, thereby posing certain limitations when applied to predict the condition of RM patients. Our novel model, tailored for RM patients, exhibits an AUC of 0.840, 0.842, and 0.842 for predicting 28-day, 60-day, and 90-day survival rates, respectively (compared to 0.734, 0.742, and 0.725 for the SOFA score), demonstrating its superior discriminatory ability. Through evaluations of NRI and IDI values, we have confirmed the superior performance of our new model. DCA validates the clinical utility of our model. Lastly, the nomogram is simple and easy to understand, transforming complex statistical models into intuitive graphics for easy comprehension by clinicians and patients alike. Consequently, in clinical practice, physicians can utilize this model for early risk assessment of patients, formulating more rational treatment plans and prognosis management strategies, ultimately enhancing patient survival rates and quality of life.

## 5 Limitations

This study has developed a comprehensive nomogram for predicting the prognosis of ICU-admitted patients with rhabdomyolysis, which further refines the traditional SOFA score by incorporating specific prognostic indicators, thereby enhancing its prognostic predictive capability. However, our study is not devoid of limitations. Firstly, due to the diverse etiology of rhabdomyolysis (such as drug-induced, exercise-induced, traumatic, etc.), there may be significant variations in clinical characteristics, disease progression, and prognosis among patients. This heterogeneity can not only affect the predictive accuracy of the model but also lead to inconsistent performance across different patient subgroups. Our study utilized data from a single center in the United States, which also contributes to heterogeneity and may limit the applicability of our findings to patient populations from different regions, racial backgrounds, socioeconomic statuses, or medical care levels. Future studies could conduct further stratified analyses, develop subgroup models, or introduce new variables to capture this heterogeneity. Secondly, although we screened and analyzed most variables, including patient demographics, physiological indicators, blood tests, comorbidities, and admission assessments, there may still be potential predictive variables that were not included, which could result in selection bias and impact the study results. Lastly, our model was only trained and internally validated using data from the MIMIC-IV database. Therefore, future research requires external validation based on independent datasets to further confirm the performance and accuracy of this novel nomogram.

## 6 Conclusion

In summary, we have developed a predictive model for assessing the survival rates of patients with rhabdomyolysis, which exhibits excellent predictive value for 28-day, 60-day, and 90-day survival outcomes among RM patients. Notably, our model outperforms the SOFA score in terms of prediction accuracy. Future research should aim to further validate this model in different patient cohorts.

## Figures and Tables

**Table:** 

RM	rhabdomyolysis
Hb	hemoglobin
PLT	platelet count
WBC	white blood cell count
RDW	red cell distribution width
AG	anion gap
HCO3	carbonic acid hydrogen radical
BUN	blood urea nitrogen
Cr	creatinine
ALT	alanine aminotransferase
ALP	alkaline phosphatase
AST	aspartate aminotransferase
TBIL	total bilirubin
PT	prothrombin time
APTT	activated partial thromboplastin time
CK	creatine kinase
Ca	calcium
K	potassium
HR	heart rate
SBP	systolic blood pressure
DBP	diastolic blood pressure
RR	respiratory rate
SPO2	oxygen saturation
C-index	concordance index
AUC	area under the receiver operating characteristic curve
DCA	decision curve analysis
NRI	net reclassification improvement
IDI	integrated discrimination improvement

## Data Availability

Publicly available datasets were analyzed in this study. The data were available on the MIMIC-IV website at https://mimic-iv.mit.edu/.

## Footnotes

^1^ https://mimic.physionet.org/

## References

[1] Cabrera-Serrano M Ravenscroft G. Recent advances in our understanding of genetic rhabdomyolysis. Curr Opin Neurol. (2022) 35:651–7. doi: 10.1097/WCO.0000000000001096, PMID: 35942668

[2] Antons KA Williams CD Baker SK Phillips PS. Clinical perspectives of statin-induced rhabdomyolysis. Am J Med. (2006) 119:400–9. doi: 10.1016/j.amjmed.2006.02.007, PMID: 16651050

[3] Huerta-Alardín AL Varon J Marik PE. Bench-to-bedside review: rhabdomyolysis – an overview for clinicians. Crit Care. (2005) 9:158–69. doi: 10.1186/cc2978, PMID: 15774072 PMC1175909

[4] Mastaglia FL Needham M. Update on toxic myopathies. Curr Neurol Neurosci Rep. (2012) 12:54–61. doi: 10.1007/s11910-011-0232-9, PMID: 21968786

[5] Sinert R Kohl L Rainone T Scalea T. Exercise-induced rhabdomyolysis. Ann Emerg Med. (1994) 23:1301–6. doi: 10.1016/s0196-0644(94)70356-6, PMID: 8198305

[6] Stahl K Rastelli E Schoser B. A systematic review on the definition of rhabdomyolysis. J Neurol. (2020) 267:877–82. doi: 10.1007/s00415-019-09185-4, PMID: 30617905

[7] Zimmerman JL Shen MC. Rhabdomyolysis. Chest. (2013) 144:1058–65. doi: 10.1378/chest.12-2016, PMID: 24008958

[8] Nathan N. Rhabdomyolysis and hepatic injury. Anesth Analg. (2023) 136:841. doi: 10.1213/ANE.0000000000006482, PMID: 37058719

[9] McMahon GM Zeng X Waikar SS. A risk prediction score for kidney failure or mortality in rhabdomyolysis. JAMA Intern Med. (2013) 173:1821–8. doi: 10.1001/jamainternmed.2013.9774, PMID: 24000014 PMC5152583

[10] De Meijer AR Fikkers BG De Keijzer MH Van Engelen BGM Drenth JPH. Serum creatine kinase as predictor of clinical course in rhabdomyolysis: a 5-year intensive care survey. Intensive Care Med. (2003) 29:1121–5. doi: 10.1007/s00134-003-1800-5, PMID: 12768237

[11] Better OS Abassi ZA. Early fluid resuscitation in patients with rhabdomyolysis. Nat Rev Nephrol. (2011) 7:416–22. doi: 10.1038/nrneph.2011.56, PMID: 21587227

[12] Chatzizisis YS Misirli G Hatzitolios AI Giannoglou GD. The syndrome of rhabdomyolysis: complications and treatment. Eur J Intern Med. (2008) 19:568–74. doi: 10.1016/j.ejim.2007.06.037, PMID: 19046720

[13] Baxter RE Moore JH. Diagnosis and treatment of acute exertional rhabdomyolysis. J Orthop Sports Phys Ther. (2003) 33:104–8. doi: 10.2519/jospt.2003.33.3.104 12683685

[14] Iasonos A Schrag D Raj GV Panageas KS. How to build and interpret a nomogram for Cancer prognosis. JCO. (2008) 26:1364–70. doi: 10.1200/JCO.2007.12.9791, PMID: 18323559

[15] Johnson AEW Bulgarelli L Shen L Gayles A Shammout A Horng S. MIMIC-IV, a freely accessible electronic health record dataset. Sci Data. (2023) 10:1. doi: 10.1038/s41597-022-01899-x, PMID: 36596836 PMC9810617

[16] Johnson A Bulgarelli L Pollard T Horng S Celi LA Mark R. MIMIC-IV (version 2.0). PhysioNet. (2022). doi: 10.13026/7vcr-e114

[17] Melli G Chaudhry V Cornblath DR. Rhabdomyolysis: an evaluation of 475 hospitalized patients. Medicine. (2005) 84:377–85. doi: 10.1097/01.md.0000188565.48918.41 16267412

[18] Clarkson PM Kearns AK Rouzier P Rubin R Thompson PD. Serum Creatine kinase levels and renal function measures in exertional muscle damage. Med Sci Sports Exerc. (2006) 38:623–7. doi: 10.1249/01.mss.0000210192.49210.fc, PMID: 16679975

[19] Lüdtke O Robitzsch A Grund S. Multiple imputation of missing data in multilevel designs: a comparison of different strategies. Psychol Methods. (2017) 22:141–65. doi: 10.1037/met0000096, PMID: 27607544

[20] Klein JP Rizzo JD Zhang MJ Keiding N. Statistical methods for the analysis and presentation of the results of bone marrow transplants. Part 2: regression modeling. Bone Marrow Transplant. (2001) 28:1001–11. doi: 10.1038/sj.bmt.1703271, PMID: 11781608

[21] Park SY. Nomogram: an analogue tool to deliver digital knowledge. J Thorac Cardiovasc Surg. (2018) 155:1793. doi: 10.1016/j.jtcvs.2017.12.107, PMID: 29370910

[22] Labarère J Renaud B Fine MJ. How to derive and validate clinical prediction models for use in intensive care medicine. Intensive Care Med. (2014) 40:513–27. doi: 10.1007/s00134-014-3227-6, PMID: 24570265

[23] Han D Xu F Li C Zhang L Yang R Zheng S. A novel nomogram for predicting survival in patients with severe acute pancreatitis: an analysis based on the large MIMIC-III clinical database. Emer Med Int. (2021) 2021:1–12. doi: 10.1155/2021/9190908, PMID: 34676117 PMC8526213

[24] Delaney KA Givens ML Vohra RB. Use of RIFLE criteria to predict the severity and prognosis of acute kidney injury in emergency department patients with rhabdomyolysis. J Emerg Med. (2012) 42:521–8. doi: 10.1016/j.jemermed.2011.03.008, PMID: 21549548

[25] Zutt R van der Kooi AJ Linthorst GE Wanders RJA de Visser M. Rhabdomyolysis: review of the literature. Neuromuscul Disord. (2014) 24:651–9. doi: 10.1016/j.nmd.2014.05.005, PMID: 24946698

[26] McKenna MC Kelly M Boran G Lavin P. Spectrum of rhabdomyolysis in an acute hospital. Ir J Med Sci. (2019) 188:1423–6. doi: 10.1007/s11845-019-01968-y, PMID: 30680491

[27] Liu C Yuan Q Mao Z Hu P Wu R Liu X. Development and validation of a model for the early prediction of the RRT requirement in patients with rhabdomyolysis. Am J Emerg Med. (2021) 46:38–44. doi: 10.1016/j.ajem.2021.03.006, PMID: 33714053

[28] Liu C Liu X Mao Z Hu P Li X Hu J. Interpretable machine learning model for early prediction of mortality in ICU patients with rhabdomyolysis. Med Sci Sports Exerc. (2021) 53:1826–34. doi: 10.1249/MSS.0000000000002674, PMID: 33787533

[29] Bruno RR Wernly B Bagshaw SM Van Den Boogaard M Darvall JN De Geer L. The clinical frailty scale for mortality prediction of old acutely admitted intensive care patients: a meta-analysis of individual patient-level data. Ann Intensive Care. (2023) 13:37. doi: 10.1186/s13613-023-01132-x, PMID: 37133796 PMC10155148

[30] Thongprayoon C Cheungpasitporn W Mao MA Sakhuja A Erickson SB. Admission hyperphosphatemia increases the risk of acute kidney injury in hospitalized patients. J Nephrol. (2018) 31:241–7. doi: 10.1007/s40620-017-0442-6, PMID: 28975589

[31] Haider DG Lindner G Wolzt M Ahmad SS Sauter T Leichtle AB. Hyperphosphatemia is an independent risk factor for mortality in critically ill patients: results from a cross-sectional study. PLoS One. (2015) 10:e0133426. doi: 10.1371/journal.pone.0133426, PMID: 26252874 PMC4529074

[32] Hedjoudje A Farha J Cheurfa C Grabar S Weiss E Badurdeen D. Serum phosphate is associated with mortality among patients admitted to ICU for acute pancreatitis. United European Gastroenterol J. (2021) 9:534–42. doi: 10.1002/ueg2.12059, PMID: 33951327 PMC8259433

[33] Kim BK Kim CY Kim S Kim YJ Lee SH Kim JH. Associations between phosphate concentrations and hospital mortality in critically ill patients receiving mechanical ventilation. J Clin Med. (2022) 11:1897. doi: 10.3390/jcm11071897, PMID: 35407502 PMC8999466

[34] Eddington H Hoefield R Sinha S Chrysochou C Lane B Foley RN. Serum phosphate and mortality in patients with chronic kidney disease. Clin J Am Soc Nephrol. (2010) 5:2251–7. doi: 10.2215/CJN.00810110, PMID: 20688884 PMC2994087

[35] Kestenbaum B Sampson JN Rudser KD Patterson DJ Seliger SL Young B. Serum phosphate levels and mortality risk among people with chronic kidney disease. J Am Soc Nephrol. (2005) 16:520–8. doi: 10.1681/ASN.2004070602, PMID: 15615819

[36] Loughlin PC Sebat F Kellett JG. Respiratory rate: the forgotten vital sign-make it count! Jt Comm J Qual Patient Saf. (2018) 44:494–9. doi: 10.1016/j.jcjq.2018.04.014 30071969

[37] Buist M Bernard S Nguyen TV Moore G Anderson J. Association between clinically abnormal observations and subsequent in-hospital mortality: a prospective study. Resuscitation. (2004) 62:137–41. doi: 10.1016/j.resuscitation.2004.03.005, PMID: 15294398

[38] Churpek MM Adhikari R Edelson DP. The value of vital sign trends for detecting clinical deterioration on the wards. Resuscitation. (2016) 102:1–5. doi: 10.1016/j.resuscitation.2016.02.005, PMID: 26898412 PMC4834231

[39] Cretikos MA Bellomo R Hillman K Chen J Finfer S Flabouris A. Respiratory rate: the neglected vital sign. Med J Aust. (2008) 188:657–9. doi: 10.5694/j.1326-5377.2008.tb01825.x, PMID: 18513176

[40] Ferreira FL Bota DP Bross A Mélot C Vincent JL. Serial evaluation of the SOFA score to predict outcome in critically ill patients. JAMA. (2001) 286:1754–8. doi: 10.1001/jama.286.14.1754, PMID: 11594901

[41] Raux M Thicoïpé M Wiel E Rancurel E Savary D David J-S. Comparison of respiratory rate and peripheral oxygen saturation to assess severity in trauma patients. Intensive Care Med. (2006) 32:405–12. doi: 10.1007/s00134-005-0063-8, PMID: 16485093

[42] Bellville JW Howland WS. Prognosis after severe hypoxia in man. Anesthesiology. (1957) 18:389–97. doi: 10.1097/00000542-195705000-00004, PMID: 13425048

