# Supplementary material for: Predictive model for assessing the prognosis of rhabdomyolysis patients in the intensive care unit
Source: Front Med (Lausanne). 2025 Jan 10;11:1518129. doi: 10.3389/fmed.2024.1518129 (PMC11759279; doi:10.3389/fmed.2024.1518129)
Supplement: Supplementary file 1 [file Data_Sheet_1.doc]

Tabel.S1:Missing number (%) for characteristics

| Characteristic | Missing number (%) |
| --- | --- |
| Gender | 0(0) |
| Age | 0(0) |
| Weight | 12(1.6) |
| Hb min | 2(0.2) |
| PLT min | 1(0.1) |
| WBC max | 1(0.1) |
| RDW | 14(1.9) |
| AG max | 0(0) |
| HCO3 min | 0(0) |
| BUN max | 0(0) |
| Cr max | 0(0) |
| ALT max | 91(12.5) |
| ALP max | 92(12.6) |
| AST max | 88(12.1) |
| TBIL max | 94(12.9) |
| PT max | 41(5.6) |
| APTT max | 48(6.6) |
| CK max | 32(4.4) |
| Ca min | 3(0.4) |
| Ca max | 3(0.4) |
| Phosphate max | 21(2.8) |
| K max | 0(0) |
| HR mean | 3(0.4) |
| SBP mean | 8(1.1) |
| DBP mean | 8(1.1) |
| RR mean | 5(0.6) |
| SPO2 mean | 4(0.5) |
| Blood glucose mean | 5(0.6) |
| Urineoutput | 10(1.3) |
| Hyperlipidemia | 0(0) |
| Hypertension | 0(0) |
| Chronic pulmonary disease | 0(0) |
| Liver disease | 0(0) |
| Diabetes | 0(0) |
| Renal disease | 0(0) |
| Hypothyroidism | 0(0) |
| SOFA | 0(0) |

Table.S2: Descriptive statistical analysis of data before and after imputation

| **Characteristic** | **Before Imputation Data**, N = 725 | **After Imputation Data**, N = 725 | **p-value** |
| --- | --- | --- | --- |
| Weight | 81.60 (69.40, 98.00) | 81.90 (69.50, 98.30) | 0.919 |
| Hb min | 11.00 (9.40, 12.40) | 11.00 (9.40, 12.40) | 0.948 |
| PLT min | 164.50 (110.00, 215.25) | 165.00 (110.00, 216.00) | 0.970 |
| WBC max | 13.90 (10.20, 18.00) | 13.90 (10.20, 18.00) | 0.972 |
| RDW | 14.00 (13.20, 15.00) | 14.00 (13.20, 15.00) | 0.923 |
| ALT max | 76.50 (39.00, 272.50) | 74.00 (38.00, 232.00) | 0.403 |
| ALP max | 76.00 (58.00, 104.00) | 76.00 (58.00, 104.00) | 0.998 |
| AST max | 187.00 (81.00, 668.00) | 171.00 (77.00, 539.00) | 0.364 |
| TBIL max | 0.70 (0.40, 1.30) | 0.70 (0.40, 1.20) | 0.676 |
| PT max | 13.80 (12.50, 16.90) | 13.70 (12.40, 16.70) | 0.828 |
| PTT max | 31.30 (27.20, 41.00) | 31.10 (27.20, 41.00) | 0.934 |
| CK max | 5,451.00 (1,888.00, 14,603.00) | 5,136.00 (1,883.00, 14,443.00) | 0.728 |
| Ca min | 7.60 (7.00, 8.20) | 7.60 (7.00, 8.20) | 0.993 |
| Ca max | 8.40 (7.90, 8.90) | 8.40 (7.90, 8.90) | 0.973 |
| P max | 4.00 (3.10, 5.40) | 4.00 (3.10, 5.40) | 0.818 |
| HR mean | 90.00 (80.00, 102.75) | 90.00 (80.00, 103.00) | >0.999 |
| SBP mean | 117.00 (107.00, 131.00) | 117.00 (107.00, 131.00) | 0.915 |
| DBP mean | 66.00 (59.00, 75.00) | 66.00 (59.00, 75.00) | 0.958 |
| RR mean | 20.00 (17.00, 23.00) | 20.00 (17.00, 23.00) | 0.976 |
| SPO2 mean | 97.00 (96.00, 99.00) | 97.00 (96.00, 99.00) | 0.992 |
| Blood glucose mean | 125.20 (102.60, 155.45) | 125.20 (102.50, 155.60) | 0.978 |
| Urineoutput | 1,635.00 (773.50, 2,783.00) | 1,635.00 (770.00, 2,780.00) | 0.926 |

Table.S3: Baseline characteristics and outcomes in the training cohort

| Characteristic | Survival, N = 403^1^ | Nonsurvival, N = 104^1^ | p-value^2^ |
| --- | --- | --- | --- |
| Gender |  |  | 0.904 |
| Male | 283 (70.22%) | 74 (71.15%) |  |
| Female | 120 (29.78%) | 30 (28.85%) |  |
| Age | 53.00 (39.00, 65.50) | 65.50 (51.00, 78.00) | <0.001 |
| Weight | 83.40 (70.20, 99.10) | 83.50 (68.30, 99.13) | 0.441 |
| Hb min | 11.00 (9.45, 12.40) | 10.60 (8.48, 12.03) | 0.035 |
| PLT min | 168.00 (121.50, 215.50) | 130.50 (75.00, 183.00) | <0.001 |
| WBC max | 13.60 (9.90, 18.00) | 15.65 (11.90, 21.25) | 0.003 |
| RDW | 13.90 (13.20, 14.80) | 14.15 (13.30, 16.33) | 0.006 |
| AG max | 18.00 (15.00, 22.00) | 21.00 (17.00, 26.25) | <0.001 |
| HCO3 min | 20.00 (17.00, 22.00) | 17.00 (13.00, 21.00) | <0.001 |
| BUN max | 25.00 (15.00, 44.00) | 35.00 (25.00, 56.75) | <0.001 |
| Cr max | 1.60 (0.90, 2.90) | 2.20 (1.40, 3.80) | <0.001 |
| ALT max | 71.00 (38.00, 182.00) | 137.50 (48.00, 561.25) | <0.001 |
| ALP max | 72.00 (56.00, 92.50) | 96.50 (69.00, 143.25) | <0.001 |
| AST max | 166.00 (76.00, 468.50) | 371.00 (122.50, 1,291.50) | <0.001 |
| TBIL max | 0.70 (0.40, 1.10) | 1.00 (0.50, 2.20) | <0.001 |
| PT max | 13.50 (12.40, 15.85) | 16.25 (13.58, 24.28) | <0.001 |
| APTT max | 30.60 (27.05, 38.15) | 41.60 (31.70, 92.60) | <0.001 |
| CK max | 5,531.00 (2,106.50, 14,810.50) | 4,297.00 (1,280.75, 11,688.00) | 0.048 |
| Ca min | 7.60 (7.00, 8.20) | 7.55 (6.80, 8.10) | 0.485 |
| Ca max | 8.30 (7.90, 8.90) | 8.50 (7.80, 8.93) | 0.678 |
| Phosphate max | 3.80 (3.00, 5.15) | 4.60 (3.58, 7.43) | <0.001 |
| K max | 4.50 (4.00, 5.30) | 5.00 (4.30, 5.90) | 0.001 |
| HR mean | 89.00 (80.00, 101.50) | 90.00 (82.75, 102.25) | 0.469 |
| SBP mean | 118.00 (108.00, 132.00) | 112.00 (103.00, 123.25) | <0.001 |
| DBP mean | 66.00 (60.00, 76.00) | 62.00 (55.00, 72.00) | 0.002 |
| RR mean | 19.00 (17.00, 22.00) | 22.00 (18.00, 26.00) | <0.001 |
| SPO2 mean | 98.00 (96.00, 99.00) | 97.00 (95.00, 98.00) | <0.001 |
| Blood glucose mean | 123.40 (101.55, 150.35) | 141.35 (117.85, 173.38) | <0.001 |
| Urineoutput | 1,820.00 (947.50, 2,950.00) | 754.50 (352.25, 1,837.50) | <0.001 |
| Hyperlipidemia | 89 (22.08%) | 29 (27.88%) | 0.241 |
| Hypertension | 154 (38.21%) | 40 (38.46%) | >0.999 |
| Chronic pulmonary disease | 82 (20.35%) | 25 (24.04%) | 0.420 |
| Liver disease | 88 (21.84%) | 38 (36.54%) | 0.003 |
| Diabetes | 97 (24.07%) | 23 (22.12%) | 0.796 |
| Renal disease | 54 (13.40%) | 16 (15.38%) | 0.633 |
| Hypothyroidism | 35 (8.68%) | 11 (10.58%) | 0.566 |
| SOFA | 5.00 (3.00, 9.00) | 10.00 (6.00, 15.00) | <0.001 |
| ICU LOS | 3.00 (1.50, 7.00) | 3.50 (2.00, 8.00) | 0.264 |
| Hospital LOS | 10.00 (6.00, 18.00) | 8.00 (3.00, 13.25) | <0.001 |

Figure S1: Comparison of Data Distribution Before and After Imputation


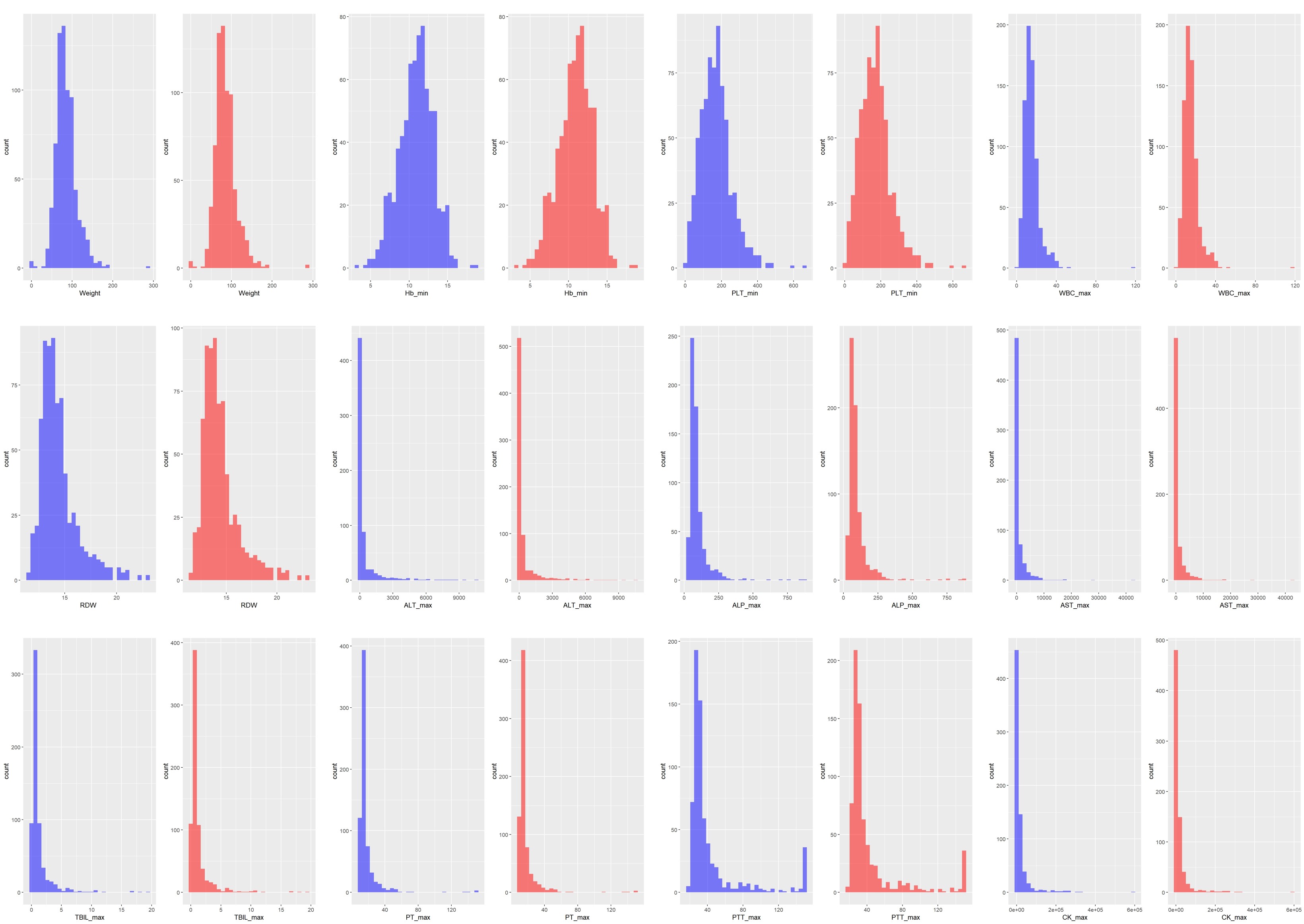


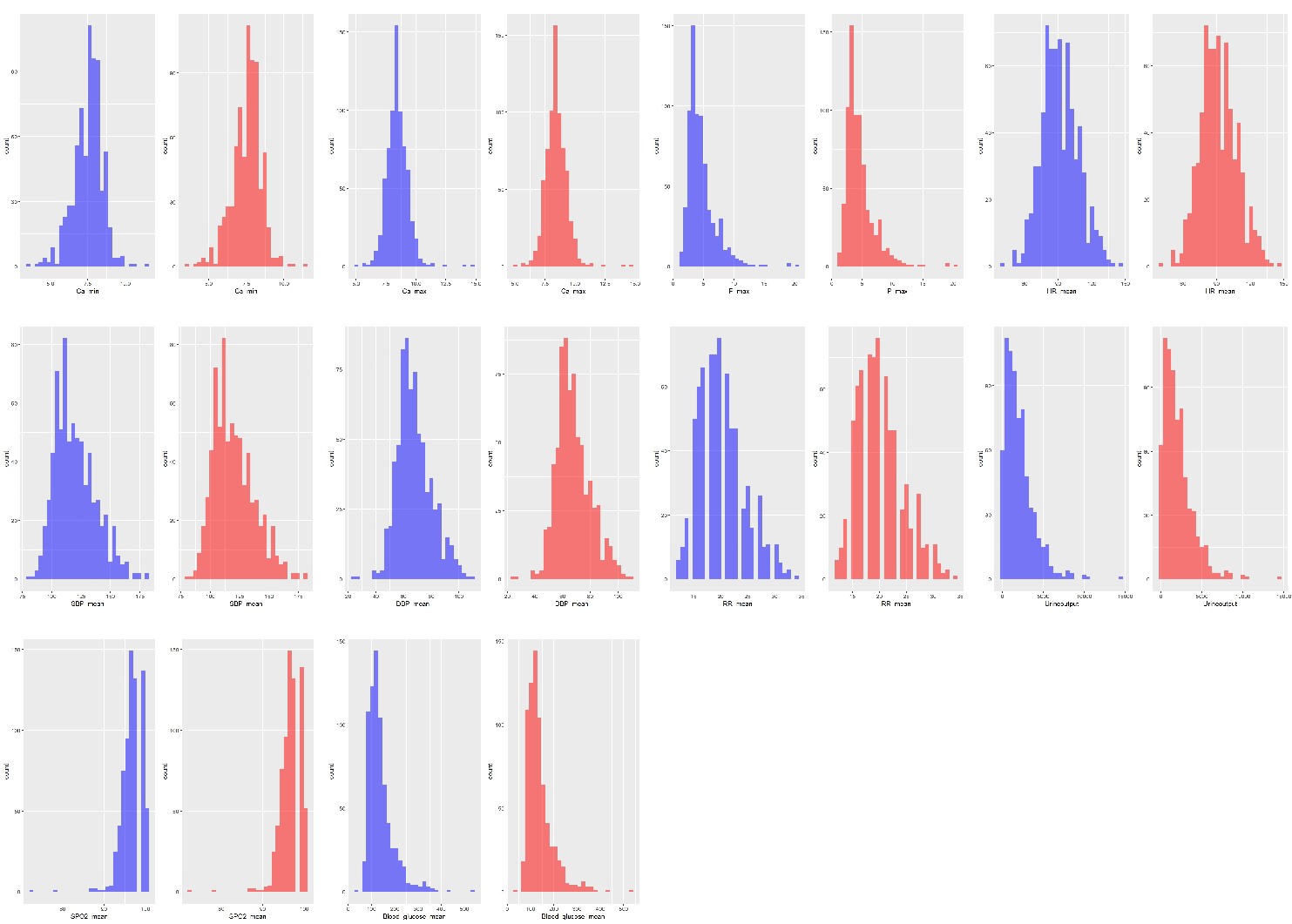

Blue: data before imputation; Red: data after imputation
